# Pain hypersensitivity in juvenile idiopathic arthritis: a quantitative sensory testing study

**DOI:** 10.1186/1546-0096-12-39

**Published:** 2014-09-06

**Authors:** Laura Cornelissen, Carolina Donado, Joseph Kim, Laura Chiel, David Zurakowski, Deirdre E Logan, Petra Meier, Navil F Sethna, Markus Blankenburg, Boris Zernikow, Robert P Sundel, Charles B Berde

**Affiliations:** Department of Anesthesiology, Perioperative and Pain Medicine, Boston Children’s Hospital; Department of Anesthesia, Harvard Medical School, 333 Longwood Avenue, Boston, MA 02115 USA; Program in Rheumatology, Division of Immunology, Department of Medicine, Boston Children’s Hospital; Department of Pediatrics, Harvard Medical School Boston, Boston, USA; Department of Psychiatry, Harvard Medical School, Boston, USA; Department of Pediatric Neurology, Psychosomatic Medicine and Pain Therapy, Center for Child and Adolescent Medicine Olgahospital, Klinikum, Stuttgart, Germany; German Paediatric Pain Centre, Children’s and Adolescents’ Hospital, Datteln; Chair of Children’s Pain Therapy and Paediatric Palliative Care, Witten/Herdecke University, Datteln, Germany

**Keywords:** Quantitative sensory testing, Children, Adolescents, Pain, Inflammation, Arthritis, Somatosensory profile, Hyperalgesia

## Abstract

**Background:**

Juvenile Idiopathic Arthritis (JIA) is the most common cause of non-infectious joint inflammation in children. Synovial inflammation results in pain, swelling and stiffness. Animal and adult human studies indicate that localized joint-associated inflammation may produce generalized changes in pain sensitivity. The aim was to characterize pain sensitivity in children with JIA to mechanical and thermal stimulus modalities using quantitative sensory testing (QST) at an affected inflamed joint, and compare to children in clinical remission. Generalized hypersensitivity was evaluated by comparing QST measures at the thenar eminence between JIA and healthy control children.

**Methods:**

60 children aged 7–17 years with JIA participated. QST assessed sensory detection threshold and pain threshold at two sites: (1) affected joint (clinically active or inactive), (2) contralateral thenar eminence. Joint site included finger, wrist, knee and ankle. Clinical status was measured using objective and subjective markers of disease severity. Questionnaires assessed pain intensity and frequency, functional disability, anxiety, pain catastrophization and fatigue. QST data collected from joints were compared within JIA patients: active vs. inactive inflammation; and data from the contralateral thenar eminence were compared between JIA and healthy control cohorts in Europe [EU, (n = 151)] and the US (n = 92). Statistical analyses were performed using Kruskal-Wallis with Dunn’s post-hoc comparison, Mann-Whitney or Fisher’s exact test, where appropriate.

**Results:**

Overall, children with JIA reported low pain scores and low degrees of functional disability. Sensory detection thresholds and pain thresholds were similar in “active” compared to “inactive” joints. Despite this, children with JIA had generalized hypersensitivity at the thenar eminence when compared to healthy children for pressure (vs. EU p < 0.001), light touch (vs. EU p < 0.001), cold (vs EU, p < 0.01; vs US, p < 0.001) and heat pain (vs EU, p < 0.05; vs US p < 0.001).

**Conclusions:**

JIA is associated with increased sensitivity to painful mechanical and thermal stimuli, even in absence of pain reports, or markers of disease activity. Future research investigating mechanisms underlying pain hypersensitivity in JIA is warranted; this will in turn guide pharmacologic and non-pharmacologic interventions to prevent or reverse these processes.

**Electronic supplementary material:**

The online version of this article (doi:10.1186/1546-0096-12-39) contains supplementary material, which is available to authorized users.

## Background

Juvenile Idiopathic Arthritis (JIA) is the most common chronic rheumatologic disease in children. It is characterized by synovial inflammation, resulting in pain, swelling, and stiffness [1, 2]. Pain has a considerable effect on the child’s well-being and is associated with sleep disturbances, functional disability, and psychosocial distress [3]. In some children with JIA whose disease is well-controlled with anti-inflammatory therapy and who have no clinical signs of joint inflammation, pain continues to be a burden [4]. Prolonged pain could be due to ongoing inflammation, or may reflect long-term changes in underlying central and peripheral sensory processing mechanisms during an important time of development in childhood [3].

Previous studies of sensory function in children with JIA have largely concerned pressure pain perception. Children with chronic inflammatory arthritis show widespread reductions in pressure pain threshold [5–7]. Even in the absence of clinical signs of joint inflammation, children with JIA who are in remission show pressure pain hypersensitivity compared to healthy controls, and this extends to non-joint areas not associated with JIA [5].

Quantitative sensory testing (QST) is a non-invasive approach used in pain research to assess large and small nerve fiber function. It is useful for assessing nociceptive (small fiber AÎ´, C) and non-nociceptive (large Aβ) mechanical sensations [8]. Thermal, mechanical touch and vibration detection thresholds, as well as cold, heat, deep pressure and mechanical pain can be assessed at multiple sites across the body. QST has been used extensively in adults, and in children, using a modified protocol that is feasible in children from 5 years of age [9–11].

Previous studies provide evidence to suggest that pressure pain hypersensitivity exists in areas unaffected by inflammation, and also in children with JIA in clinical remission [5–7]. However, there is little information regarding the complete mechanical and thermal sensory profile of children with JIA. The primary aim of this study was to examine sensory detection threshold and pain threshold in children with JIA at the site of active inflammation, at the upper extremities: fingers (distal or proximal interphalangeal joint) or wrist; or lower extremities: knee or ankle, and compare to children in clinical remission. Sensory thresholds at the contralateral thenar eminence (a remote, non-joint control site) were then compared against two sets of healthy age-matched controls cohorts from the US and Europe.

## Methods

### Study design

JIA patients were recruited from the Rheumatology Clinic at Boston Children’s Hospital during routine clinic visits from November 2012 to September 2013. Subjects underwent quantitative sensory testing (QST) at a joint and non-joint control (thenar eminence) site (see Methods- QST), after completing a series of psychological questionnaires on the day of their visit. Demographics and clinical information including age, gender, disease duration, JIA subtype and markers of disease activity were collected by the treating rheumatologist on the day of the study visit.

Children and adolescents were eligible if they were aged 7 to 17 years and diagnosed with JIA. Additional criteria were (1) English speaking; (2) absence of cognitive impairment or a known developmental disability; and (3) no evidence of a neurological disorder. Use of analgesics or non-steroidal anti-inflammatory drugs (NSAID) on the day of testing were noted for all children. No patients were taking opioid analgesia at the time of study.

Parents/guardians provided written informed consent and all patients provided written assent. The Institutional Review Board at Boston Children’s Hospital approved the study.

### Markers of disease activity and duration

Measurements for classifying the subject’s disease activity level according to the Juvenile Arthritis Disease Activity Score (JADAS-10) criteria were collected by the examining pediatric rheumatologist at each study visit [12, 13]. Parameters followed included (1) number of joints with active arthritis; (2) physician global assessment of disease activity, measured on a 10 cm visual analog scale (VAS) where 0 = no activity and 10 = maximum activity; (3) parent/patient assessment of overall well-being, measured on a 10 cm VAS scale where 0 = very well and 10 = poor; (4) functional ability (Child Health Assessment Questionnaire, C-HAQ); (5) measures of systemic inflammation (Erythrocyte Sedimentation Rate or C-reactive protein level).

### Pain experience, functional status and psychosocial factors

Pain experience (pain intensity and frequency), functional disability and mental health status were assessed using a series of self-report written questionnaires completed by subjects before performing QST.

Pain intensity on the day of study was rated on a standard numerical rating scale of 0–10, where 10 is worst pain imaginable. Frequency of pain episodes over the last year was evaluated with the following question: “Please put a circle that best describes how often you feel pain: (a) never in the past year, (b) about once a year, (c) several times a year, (d) once a month, (e) several times at month, (f) about once a week, (g) several times a week, (h) daily or almost daily”.

Functional disability was measured using the Functional Disability Inventory (FDI). The FDI is used to assess the extent of pain-related functional impairment across domains (e.g. physical and social functioning) and is a validated child-report measure [14–16]. Pain catastrophizing was measured using the child version of the Pain Catastrophizing Scale (PCS) [17, 18]. Presence of psychosocial dysfunction of daily life at home, school, with friends, in activities, and/or in mood or self-esteem was assessed using the Pediatric Symptoms Checklist [19, 20]. Trait anxiety was assessed with “trait” portion of the State Trait Anxiety Inventory for Children (T-STAIC) [21]. Pediatric rheumatology-specific quality of life for fatigue was assessed using the PedsQL Multidimensional Fatigue Scale (PedsQL), a validated and reliable instrument designed to measure general, sleep/rest and cognitive fatigue [22].

### Quantitative Sensory Test (QST)

#### Test site

QST was performed at two sites on the body: (1) an affected joint and (2) the contralateral thenar eminence. Signs of joint inflammation were identified by individual assessment from the physical examination. The “Active” joint was identified as the joint with the most significant inflammation, swelling or tenderness. For those patients who did not have an “Active” joint, an “Inactive” joint, with the most inflammation in the past was tested (as reported in the medical notes or by the patient). Joint test site was the distal or proximal interphalangeal joint, wrist, knee or ankle. The contralateral thenar eminence was the control site.

#### Protocol

The QST protocol consisted of a series of 8 sensory tests. Mechanical (MDT), vibration (VDT), cool (CDT) and warm (WDT) detection thresholds, mechanical “punctate” pain (MPT), pressure pain (PPT), cold pain (CPT) and heat pain thresholds (HPT) were assessed. We used a modified QST protocol that has been validated in healthy children and adolescents previously by our group [11, 23]. We included additional sensory tests designed to assess mechanical tactile and pressure stimulus modalities; these modalities were tested according to the recommendations published by our collaborators in Germany [9]. All thresholds were determined using the Method of Limits approach that uses stimuli of increasing intensity, starting at sub-threshold levels, to determine sensory thresholds [11]. A copy of the QST script used is available in the Additional file 1. The QST protocol was performed in the order listed below:

##### Mechanical stimuli: Mechanical detection (MDT) and Mechanical pain threshold (MPT)

A standardized set of Von Frey hairs (North Coast Medical, USA) that exert precise forces between 0.008 g and 300 g were applied perpendicularly to the skin, and the subject asked to report the sensation felt, if any. Children were asked to report when they first felt a sensation for MDT (this was repeated 3 times), and when they felt a sharp-prick like sensation for MPT (repeated 2 times at each site). Stimuli were applied at slightly different sites within a small area to avoid habituation.

##### Vibration detection threshold (VDT)

A vibrometer consisting of a small computer-controlled round plastic disc (1 cm^2^) was placed perpendicularly to the test site and the frequency of vibration was gradually increased in strength until the patient felt sensation (TSA II, Medoc Inc., Israel). The patient was asked to press a button when the vibration sensation was first felt. This test was repeated 8 times.

##### Pressure pain threshold (PPT)

A pressure algometer (FDX 50,Wagner Inc., USA) was used to quantify pressure sensation. A small flat disc with a surface area of 1.1 cm^2^ attached to a hand-held probe was held perpendicularly to the test site and gradually brought in contact with the skin. The patient was asked to report when s/he felt pressure pain (PPT). This was repeated 3 times.

##### Thermal stimuli: Cool and warm detection (CDT, WDT), cold and hot pain threshold (CPT, HPT)

A computer-operated thermode was placed on the skin and secured by a Velcro strap (TSA II, Medoc Inc., Israel). The thermode contact area measured 1.6 × 1.6 cm. The baseline temperature was 32°C and cut-off temperature limits were 0 and 50°C. For cool and warm detection assessment, the rate of temperature change was 1°C/s; for cold and heat pain threshold assessment, the rate of temperature change was 1.5°C/s. The patient was asked to identify the sensations of warmth and cool, and hot and cold by pressing a button when each sensation was first felt. Once the button was pressed, the value was automatically recorded and the thermode temperature returned to baseline at rate of 1°C/s for cool and warm sensation, and 10°C/s for cold and hot pain. Cool and warm stimuli were measured 4 times, and, hot and cold stimuli 3 times.

### Study visit information

Studies were conducted in the clinic room by a trained sensory physiologist on the day of the clinic visit. Patients were given time to adapt to the room temperature, be introduced to the questionnaires and QST equipment. Patients were seated on the clinic bed, positioned such that they could not see what was appearing on any data collection monitors, and during testing were asked to keep their eyes closed. Parent(s) or guardians were present throughout the testing.

Each study visit took an average of 40.5 min (SD = 10.1 min) to complete. We were able to maintain the subject’s concentration throughout the study. Mean room temperature was 24.7°C (SD = 2.2) and mean room humidity was 45.1% (SD = 15.7). All subjects detected mechanical pain at a higher force than mechanical detection, cold pain at a lower temperature than cool detection threshold, and hot pain at a higher temperature than warm sensation threshold. These data provide a control that the youngest children understood the verbal instructions. No subject withdrew from the protocol.

### Control groups

Previous data collected from QST studies in healthy children and adolescents were used as control groups [9, 11]. Results of QST can be influenced by the subtleties of testing protocols and testing situation. In order to make comparisons more generalizable and to account for some improvements in testing paradigms over time, we chose prospectively to include two large cohort QST data sets acquired from healthy children in the US and Germany (Europe, EU) [9, 11].

For the US controls, 5 QST parameters were evaluated at the thenar eminence and the foot in 101 subjects aged 6–17 years: vibration detection threshold (VDT), and thermal thresholds [cold detection, (CDT), warm detection (WDT), cold pain (CPT) and heat pain (HPT)]. Vibratory and thermal thresholds were evaluated using an automated vibrometer and 3×3 cm Peltier thermode (TSA II, Medoc Inc., Israel). Subjects were recruited from the hospital staffs’ children, the well-child outpatient clinic and from a local school. Healthy children with no evidence of neurological disorders were eligible for the study. Children younger than 7 years were excluded from our analysis. The final US control group included QST data from 92 of the total 101 children.

European controls were tested for 13 QST parameters from the thenar eminence, masseter and ball of the foot. A total of 167 subjects aged 6–16 years were recruited from two primary and secondary schools. QST parameters used for comparison in the present study include mechanical detection threshold (MDT), pressure pain threshold (PPT), and thermal threshold (CDT, WDT, CPT and HPT). MDT was evaluated using von Frey hair filaments (Optihar1-Set, Marstock Nervtest, Germany), PPT with a 1.1 cm^2^ tip algometer (FDX100, Wagner Instruments, USA), and thermal thresholds with a 3×3 cm Peltier thermode (TSA II, Medoc Inc., Israel). Children younger than 7 years were excluded from our analysis. The final European control group included QST data from 151 of the 167 children.

### Statistical analysis

#### Sample size calculation

The primary outcome measures were sensory threshold in JIA “Active” and “Inactive” joint groups. Availability of QST reference data available for the small joints in the pediatric population is limited. We used pressure pain threshold (PPT) as the representative measure of QST because this parameter shows consistent somatosensory abnormalities in children with chronic arthritis.

Previous studies reported JIA patients have a mean PPT of 14 N lower than healthy controls across multiple sites [5]. Mean PPT (47.6 N) and SD (17.5 N) values from the thenar eminence of EU healthy controls were taken as the reference normal. To find a mean difference of 14 N between JIA patients with active and with inactive arthritis, 52 patients (26 per group) were required to achieve a power of 80% (â€˜moderate’ effect size: 14/17.5 = 0.8; two-tailed α = 0.05; β =0.2); (nQuery v7, Statistical Solutions, MA). We enrolled patients using a convenience sample approach. Therefore, the final sample size of 60 JIA patients in the cohort provided sufficient statistical power for capturing differences that we considered significant between JIA patients with active and inactive arthritis. Secondary measures were sensory threshold changes between all JIA patients and healthy control groups, and QST predictors (see Methods- Statistics).

#### Statistics

Median, interquartile range (IQR), mean, standard deviation (SD), are given as appropriate. Thermal threshold values (CDT, WDT, CPT, HPT) are given as change from baseline thermode temperature of 32°C, and absolute values where indicated. All data were tested for normality using the D'Agostino & Pearson omnibus test and followed a non-parametric distribution. For QST, we were unable to collect data in some children with JIA for vibration (n = 7), thermal (n = 4) and pressure (n = 2) stimulus modalities due to technical issues; we did not substitute missing data. Psychological questionnaires were excluded from the final analysis if a child did not complete more than 85% questions. For partially completed psychological questionnaires, the mean score for the individual was used as a substitute value for unanswered questions. All statistical analyses were performed with GraphPad Prism 5.04 (GraphPad Software, Inc, USA) and STATA (StataCorp LP, USA).

##### Comparison within JIA patients

JIA patients were divided into two cohorts according to the location of the joint tested: (1) upper extremities (distal or interphangeal joint, or wrist); or (2) lower extremities (knee or ankle). Each cohort was subdivided into two groups depending on the clinical inflammatory status of the joint evaluated: “Active” or “Inactive” joint. Within-cohort comparisons were performed to evaluate differences sensory detection and pain thresholds at the joint test-site. All four JIA groups (inclusive of location of test joint and inflammatory status) were compared to evaluate differences in sensory detection and pain thresholds at the control site. Groups were compared with Kruskal-Wallis tests with Dunn’s correction, or Fisher’s exact test, where appropriate.

##### QST predictors

Multiple linear regression analyses of the evaluated joint were applied for each QST measure. We aimed to evaluate the association of demographic variables and other clinical factors, besides the inflammatory state of the joint, on QST threshold. We created a general model for each QST parameter that included potential variables associated with the change in sensory thresholds and pain [24]. The explanatory variables included in the model were test site, age, gender, markers of disease activity, and psychological questionnaire scores. Results are expressed as R2 of the model, the adjusted β coefficient in each predictor value, and significance level. To correct for multiple comparisons, two-tailed p < 0.01 with Bonferroni correction were considered significant.

##### Comparison between JIA and controls

For comparison of sensory detection thresholds and pain thresholds in JIA patients versus healthy controls, QST data were taken from the thenar eminence. Each QST parameter tested in the JIA group was compared to the available QST parameter of each separate control group. Mann–Whitney testing was used for these comparisons, assuming significance level less than 0.05..

## Results

### Subjects

#### JIA patients

Demographics and clinical characterization of the JIA patients are shown in Table 1. A total of 60 JIA patients, 44 girls and 16 boys (median, 13.0 years; IQR: 9.6-15.5) participated in this study. There was no difference in age between girls and boys (p = 0.76). The main JIA subtypes were polyarticular (48%) and psoriatic (22%). Sixty-eight percent of children enrolled into the study were taking disease-modifying agents at the time of testing (41/60). Methotrexate was most commonly taken (55% total subjects), followed by etanercept (27%), infliximab (10%), lefluomide (3%) and sulfasalazine (1.6%); Table 1. Eight children were taking daily NSAIDs at the time of study (2 with active arthritis, and 6 in clinical remission).

**Table 1:** **Age, gender and clinical characteristics of children with JIA** ^a^

	JIA patients			
		QST joint status		
Characteristics	Total (n = 60)	Active (n = 23)	Inactive (n = 37)	***p-value*** ^***b***^
Female patients, n (%)	44 (73%)	17 (74%)	27 (73%)	0.94^c^
Age at study visit, years	13.0 (9.6-15.5)	10.6 (9.4-15.0)	14.4 (9.7-15.9)	0.22
Duration of rheumatology clinic attendance, years, mean (SD)	5.8 (3.9)	4.9 (4.0)	6.4 (3.8)	0.17^d^
**Markers of disease activity**				
Physician's global assessment of overall disease activity^e^	1.0 (0.0-2.0)	2.0 (1.0-3.0)	0.7 (0.0-1.1)	**<0.01**
No. of active joints	1.0 (0.0-3.0)	3.0 (2.0-9.0)	0.0 (0.0-0.5)	**<0.001**
Patient's pain assessment^f^	0.0 (0.0-2.0)	2.0 (0.0-3.5)	0.0 (0.0-2.0)	0.21
Patient's global assessment of overall wellbeing^g^	1.0 (0.0-2.7)	1.2 (0.6-2.8)	0.0 (0.0-2.9)	0.10
C-HAQ score^h^	0.0 (0.0-0.13)	0.0 (0.0-0.25)	0.0 (0.0-0.0)	0.13
Erythrocyte sedimentation rate, mm/hour^i^	8.0 (4.0-11.0)	8.5 (5.0-12.5)	8.0 (3.0-11.0)	0.37
C-reactive protein level, mg/L^j^	0.1 (0.04-0.2)	0.1 (0.07-0.26)	0.1 (0.04-0.2)	0.77
**Medications (n)**				
Methotrexate	33 (55%)	14	19	
Etanercept	16 (27%)	6	10	
Infliximab	6 (10%)	1	5	
Leflunomide	2 (3%)	0	2	
Sulfasalazine	1 (1.6%)	0	1	

C-HAQ: Child Health Assessment Questionnaire; IQR- Inter-Quartile Range; QST- Quantitative Sensory Testing; SD, Standard deviation.

^a.^All values given as median (IQR) unless otherwise stated. ^b.^ *p*-values indicate differences in JIA patients with active compared to clinically inactive joints. Mann–Whitney test used unless otherwise stated. Boldface highlights significant differences. ^c.^Fisher’s Exact test. ^d.^Unpaired Student’s t-test. ^e.^Data available for 29 patients (Active, n = 7; Inactive, n = 22). ^f.^Data available for 55 patients (Active, n = 21; Inactive, n = 34). ^g.^Measured on a 100 mm visual analogue scale (0, best; 10, worst). Data available for 43 patients: Active, n = 17; Inactive, n = 26) ^h.^Data available for 45 patients (Active, n = 17; Inactive, n = 28). ^i.^Data available for 51 patients (Active, n = 20; Inactive, n = 31). ^j.^Data available for 53 patients (Active, n = 21; Inactive, n = 32).

Age, gender and duration of rheumatology clinic attendance were comparable between the “Active” joint and “Inactive” joint JIA groups (Table 1). Overall, markers of disease activity were low across the JIA patients. Physician global assessment and number of active joints were significantly different between subgroups (p < 0.01 and p < 0.001 respectively; Table 1).

Pain scores were low across the entire JIA sample. The “Active” joint group showed a trend towards higher pain scores, rating in the mild intensity range (2.0, IQR: 0.0-3.5); this was not significantly different to the “Inactive” group (vs. 0.0, IQR: 0.0-2.0; p = 0.10). Subjects in the “Active” joint group experienced more frequent pain episodes, where 67% of subjects reported pain occurring at least once a week, compared to only 32% in the “Inactive” joint group (Fisher’s exact test, p = 0.02).

Fifty-four patients completed all 5 questionnaires (4 children did not complete any questionnaires, 1 child completed 3/5, and 1 child completed 4/5). Collectively, scores for functional and psychological measures for JIA patients were within normative ranges. Functional disability (FDI) scores were rated in the lowest quartile, indicating no or minimal levels of overall disability (median score 1.0; IQR: 0.0-7.0). Trait anxiety and pain catastrophizing were evaluated with the T-STAIC and PCS questionnaires, with median scores of 26.0 (IQR: 22–33) out of 60, and 8 (IQR: 3–16) out of 64 respectively. Pediatric Symptoms Checklist scores were 9.5 (IQR 3.0-15.0) out of 70, with a score >28 being the cut-off for psychological impairment. In PedsQL fatigue assessment a high score indicates little impairment of quality of life due to fatigue. In the study sample, median PedsQL score was 88.0% (IQR: 66.0-96.0) which reflects little impact of fatigue on quality of life [22]. One child with JIA scored 11% on the PedsQL fatigue assessment, and this child had high scores in measures of functional disability (FDI: 21/60) and moderate scores in trait anxiety (T-STAIC: 31/60), pain catastrophizing scale (PCS: 13/64), pediatric symptoms checklist (PSC: 23/70), but was not an outlier in any of the pain perception or sensory threshold QST measurements.

#### Healthy controls

Details of the control groups are described in the Methods. Ages of children with JIA were comparable to healthy US controls (US controls: 13.0 years, IQR: 10.0-13.8; p = 0.26). There was a difference in age between children with JIA and healthy EU controls, but the median values lay in close range (EU controls: 11.0 years, IQR: 9.0-14.0; p < 0.05). For both the healthy US and EU controls, 50% of subjects were female, and in the JIA cohort this was 73% female.

### Comparison of sensory detection threshold and pain threshold with joint inflammation status

Table 2 summarizes the sensory detection threshold and pain threshold data at the joint test-site in children with JIA according to inflammation status of the joint (i.e. “Active” or ““Inactive”) and location of QST testing site (i.e. upper or lower extremity). All 60 JIA patients enrolled into the study completed MDT and MPT tests; we have missing values for PPT (n = 2), VDT (n = 7) and thermal tests (n = 4) due to technical failure.

**Table 2:** **Absolute QST values at joint test-site in children with JIA according to joint inflammation status** ^a^

	Upper extremity					Lower extremity				
QST parameter	Active joint (n = 11)		Inactive joint (n = 20)		***p-value*** ^***b***^	Active joint (n = 12)		Inactive joint (n = 17)		***p-value*** ^***b***^
**Innocuous stimulus**										
MDT (g)	0.05	(0.04-0.16)	0.08	(0.04-0.40)	0.11	0.28	(0.07-0.43)	0.40	(0.18-0.53)	0.26
VDT (μm/sec)	0.69	(0.54-0.84)	0.64	(0.57-0.81)	0.87	2.01	(1.74-3.61)	4.25	(2.14-5.35)	0.21
CDT (°C)	30.1	(28.2-30.7)	30.4	(29.0-30.9)	0.83	28.5	(26.2-29.1)	27.8	(24.5-29.4)	0.78
WDT (°C)	34.7	(34.3-38.5)	34.5	(33.9-35.9)	0.69	37.0	(36.1-39.2)	36.9	(35.7-38.5)	0.87
**Noxious stimulus**										
MPT (g)	0.40	(0.06-1.70)	0.50	(0.40-3.50)	0.17	0.60	(0.34-1.00)	1.00	(0.40-1.60)	0.40
PPT (N)	7.47	(5.37-15.0)	10.4	(6.50-16.3)	0.27	12.6	(10.3-21.2)	14.9	(7.78-21.6)	0.85
CPT (°C)	29.4	(21.6-30.3)	25.6	(20.0-28.6)	0.06	24.0	(16.9-26.9)	24.6	(20.4-26.0)	0.87
HPT (°C)	36.7	(35.9-42.9)	38.2	(36.3-40.8)	0.62	43.9	(40.7-45.0)	41.3	(39.1-43.2)	0.19

CDT- Cold Detection Threshold, CPT, Cold Pain Threshold; HPT, Heat Pain Threshold; IQR- Inter-Quartile Range; MDT- Mechanical Detection Threshold; MPT- Mechanical Pain Threshold; PPT, Pressure Detection Threshold; VDT- Vibration Detection Threshold; WDT- Warm Detection Threshold.

^a.^All values given as median (IQR) unless otherwise stated. Eight children with JIA were taking daily NSAIDs at the time of study. Two had active arthritis and were evaluated for QST at the finger or knee; 6 were in clinical remission and were evaluated at the finger (n = 3), knee (n = 1) or ankle (n = 1).^b.^ *p*-values indicate differences in sensory threshold between Active and Inactive joints using Mann–Whitney *U*-test.

#### Pain threshold

No significant group differences were found at the “Active” joint and the “Inactive” joint for pressure pain threshold (upper extremity, p = 0.27; lower extremity, p = 0.85), or for mechanical pain threshold (upper extremity, p = 0.17; lower extremity, p = 0.40). Thermal sensitivity was not different for cold pain threshold (upper extremity, p = 0.06; lower extremity, p = 0.87), or heat pain threshold (upper extremity, p = 0.62; lower extremity, p = 0.19) for “Active” or “Inactive” joints.

#### Sensory detection threshold

No significant group differences were observed at the “Active” joint group and the “Inactive” joint group for mechanical detection threshold (upper extremity, p = 0.11; lower extremity, p = 0.26), or for vibration detection thresholds (upper extremity, p = 0.87; lower extremity, p = 0.21). Cool detection threshold, or warm detection threshold also did not differ between joints with active or inactive inflammation (CDT: upper extremity, p = 0.83; lower extremity, p = 0.78; WDT: upper extremity, p = 0.64; lower extremity, p = 0.87).

#### Control site (thenar eminence) sensitivity

There were no significant differences in seven out of 8 sensory detection or pain threshold parameters (Table 3). Notably, cold pain was the most variable measure, and was shown to be significantly different among JIA groups (p = 0.03). Specifically, significant differences for CPT found between JIA patients with active arthritis in the upper extremity vs lower extremity after Dunn’s correction [active upper: 29.1°C (25.0-30.6); active lower: 21.1°C (15.5-25.4)].

**Table 3:** **Absolute QST values at control site (thenar eminence) in children with JIA according to joint inflammation status** ^a^

	Upper extremity				Lower extremity				
QST parameter	Active joint (n = 11)		Inactive joint (n = 20)		Active joint (n = 12)		Inactive joint (n = 17)		***p-value*** ^***b***^
**Innocuous stimulus**									
MDT (g)	0.04	(0.03-0.08)	0.04	(0.02-0.05)	0.04	(0.02-0.05)	0.05	(0.04-0.09)	0.08
VDT (μm/sec)	0.58	(0.42-1.20)	0.69	(0.59-1.03)	0.64	(0.50-0.81)	0.80	(0.63-0.91)	0.35
CDT (°C)	30.4	(29.5-30.8)	30.2	(29.7-30.7)	29.9	(29.2-30.5)	30.2	(27.5-30.6)	0.60
WDT (°C)	34.8	(33.8-36.0)	34.6	(34.2-35.2)	34.8	(34.2-36.5)	34.9	(33.7-35.5)	0.95
**Noxious stimulus**									
MPT (g)	0.8	(0.4-1.4)	1.0	(0.4-2.5)	1.2	(0.8-3.7)	1.3	(0.6-4.5)	0.46
PPT (N)	6.1	(4.5-15.0)	10.7	(8.4-16.3)	11.3	(5.8-16.9)	9.7	(7.4-15.2)	0.58
CPT (°C)	29.1	(25.0-30.6)	25.7	(14.3-29.0)	21.1	(15.5-25.4)	26.6	(22.0-28.9)	**0.03** ^**c**^
HPT (°C)	37.2	(35.7-40.3)	37.8	(35.4-43.0)	41.1	(38.6-45.5)	37.5	(35.9-43.2)	0.17

CDT- Cold Detection Threshold, CPT, Cold Pain Threshold; HPT, Heat Pain Threshold; IQR- Inter-Quartile Range; MDT- Mechanical Detection Threshold; MPT- Mechanical Pain Threshold; PPT, Pressure Detection Threshold; VDT- Vibration Detection Threshold; WDT- Warm Detection Threshold.

^a.^All values given as median (IQR) unless otherwise stated. Eight children with JIA were taking daily NSAIDs at the time of study. ^b.^ *p*-values indicate differences in sensory threshold between all four groups using Kruskal-Wallis testing. Boldface highlights significant differences. ^c.^Significant differences for CPT found between JIA patients with active arthritis in the upper extremity vs lower extremity after Dunn’s correction for multiple comparisons; no other comparisons were different.

#### QST predictors for children with JIA

We employed linear multiple regression analyses to evaluate whether demographic or clinical factors were associated with QST sensory detection thresholds or pain thresholds at the joint test-site.

Site-specific differences were found (upper extremity vs. lower extremity) for heat pain, thermal cool and warm detection, and vibration detection. Compared to the lower extremities, the upper extremities had lower sensory thresholds (increased sensitivity) to heat pain (HPT: β = 0.09, p = 0.028), thermal detection threshold (CDT: β = 0.23, p < 0.001; WDT: β = 0.17, p = 0.002) and vibration detection threshold (VDT: β = 0.35, p < 0.001). For the other QST parameters there were no other significant associations with the site tested. All other linear regression analysis included joint site tested in the models.

Hypersensitivity to thermal and mechanical stimuli was not associated with laboratory markers of disease activity (Erythrocyte Sedimentation Rate, C-reactive protein), CHAQ scores and duration of treatment for JIA (see Additional file 2: Table A).

Significant positive associations were found for the following QST variables: (1) Mechanical pain hypersensitivity (lower pain thresholds) with higher trait-anxiety scores (T-STAIC) (β = 0.12, p = 0.013); (2) cold pain hypersensitivity with females (β = 0.22, p < 0.001); and (3) cool detection hypersensitivity with higher PedsQL fatigue scores (β = 0.32, p = 0.012).

### Comparison of sensory detection threshold and pain threshold between children with JIA and healthy controls

We evaluated sensory detection and pain thresholds in children with JIA at the thenar eminence, and compared to healthy control dataset(s). There was no visible local inflammation at the thenar eminence in any child with JIA. Data from the children with JIA were pooled.

### Pain threshold

**Figure 1:**
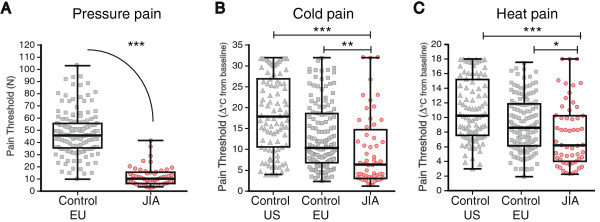
**Noxious thresholds for pressure, cold and heat pain at thenar eminence.** Pressure, and temperature change from baseline, where the sensation of **(A)** pressure, **(B)** cold and **(C)** heat pain were perceived are plotted for JIA patients (JIA, n = 58), and controls (EU, n = 151; US, n = 92). JIA patients were hypersensitive to all modalities. MPT is not included because we do not have comparison values from healthy controls. Individuals represented by single dots, Box and Whisker plot illustrates median, IQR and range. *p < 0.05; **p < 0.01; ***p < 0.001**.**

Figure 1 illustrates the differences in pain threshold between JIA patients and healthy controls for pressure and thermal stimulus modalities. Children with JIA were hypersensitive (lower pain thresholds) to pressure and thermal pain compared to healthy controls.

Children with JIA were hypersensitive to pressure stimulation and reported pain at lower median forces of 10.3 N (IQR: 6.3-15.5) compared to EU controls, 45.8 N (IQR: 35.6-55.6; p < 0.001). The differences between the groups were equivalent to at least 30 N, or 3 kg.

For thermal pain threshold, children with JIA were more sensitive to cold pain and heat pain compared to healthy controls. Children with JIA had lower sensory thresholds for cold pain (as shown by a smaller change in cold temperature from baseline), the median temperature change was 6.3°C (IQR: 3.1-14.7); US controls were 17.9°C (IQR: 10.6-26.9), and EU control were 10.4°C (IQR 6.8-18.6); (vs. US, p < 0.001; vs. EU, p < 0.01). Heat pain thresholds were evaluated in the children with JIA and compared to the same healthy control subjects, the variability within each group was smaller than for CPT. Children with JIA perceived heat pain at a median temperature increase of 6.2°C (IQR: 4.0-10.3), US control was 10.3°C (IQR: 7.6-26.9; p < 0.001), and EU control was 8.6°C (IQR: 6.1-11.9; p < 0.05). Absolute median (IQR) and p-values are given in the Additional file 3: Table B.

### Sensory detection threshold

**Figure 2:**
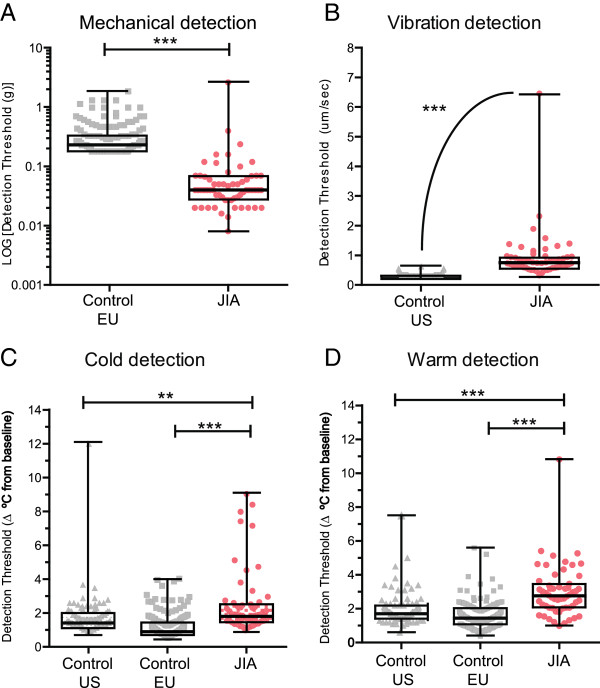
**Mechanical, vibration, cool and warmth detection thresholds at thenar eminence. (A)** Mechanical, **(B)** vibration, and **(C)** cool and **(D)** warm (temperature change from baseline), detection thresholds are plotted for JIA patients (JIA, MDT, n = 60, VDT, n = 56, CDT/WDT, n = 58), and controls (EU, n = 151; US, n = 92). JIA patients exhibited altered sensation to all innocuous modalities. Individuals represented by single dot, Box and Whisker plot illustrates median, IQR and range. **p < 0.01; ***p < 0.001.

Figure 2 illustrates sensory detection thresholds to innocuous vibration and thermal stimuli at the thenar eminence. JIA patients were more sensitive to mechanical stimuli (0.04 g, IQR 0.03-0.07) compared to healthy controls (EU 0.23 g, IQR: 0.18-0.33; p < 0.001).

In contrast, children with JIA had vibration detection thresholds of 0.68 μm/sec (IQR 0.53-0.90) that were significantly lower (hyposensitive) compared to healthy controls (US: 0.2 μm/sec, IQR: 0.2-0.25; p < 0.001). Similarly, for CDT and WDT, a greater temperature change was required for JIA patients to perceive temperature compared to healthy controls from both the US and EU (see Additional file 3: Table B).

The JIA cohort contained a higher proportion of girls (72%) compared to both healthy control data sets (50% in each). Sensitivity analyses, by excluding boys in all groups and examining QST parameters for children with JIA and healthy controls, showed no significant differences for all QST measures (data not shown).

## Discussion

We examined local and systemic inflammatory markers, current pain score, functional disability, psychological measures, and QST in children with JIA. As a group, they had mild pain scores, low disability inventories, and normal ranges for psychological measures. In addition, their local and systemic markers of inflammation or disease activity were mild. Despite these factors, children with JIA exhibited pain hypersensitivity in areas outside of the affected joint, in response to pressure, thermal cold and heat pain modalities when compared to healthy controls. Additionally, we observed that children with JIA had substantially lower sensitivity (increased thresholds) to innocuous vibratory, thermal cool and warmth modalities when compared to healthy controls. Widespread pain hypersensitivity in children with JIA suggests that potentiated nociceptive processing can occur with minimal overt evidence for ongoing inflammation.

Early sensory studies demonstrate that children with chronic arthritis and inflammation exhibit hypersensitivity to pressure pain compared to healthy controls when local pressure was applied to joint capsules at locations such as the wrists, elbows, knees, ankles and/or soft paraspinal tissues [5–7, 25]. Our data support these findings, showing generalized changes in sensitivity in children with JIA when compared to healthy controls. We also show that pain perception and sensory threshold in all mechanical and thermal stimulus modalities did not markedly differ between JIA groups with “Active” and “Inactive” joint inflammation. This result was contrary to our expectations that active joints with clinical signs of inflammation would be more sensitive than joints without signs of inflammation. Indeed, pressure pain hypersensitivity has been shown to be greater in children with active inflammation than in those with arthritis but no detectable inflammation [5]. However, other work such as that by Leegaurd et al. evaluated pressure pain in children with JIA at 17 body sites, even in areas unaffected by arthritis, and reported that pressure pain threshold was not associated with disease severity [7].

Previous epidemiologic studies and case series on pain prevalence and disease severity in JIA have varied considerably in their reported degrees of pain intensity and pain-related disability. Pain is a common experience in children with JIA, with intensity often reported in the mild to moderate range [26]. Previous studies show that higher pain scores among children with JIA correlated with lower pain pressure thresholds and pain tolerance [5, 27]. However, disease duration, disease activity and pressure pain threshold have also been shown to be independent factors in JIA [7, 27]. In our study, sensory threshold was similar in active and inactive joints. Also, since our sample were characterized by low markers of disease activity, it is plausible that subclinical signs of inflammation may have been present in some of the JIA patients allocated to the “Inactive” joint group. These differences may reflect different patient selection, secular trends over time due to improvements in disease management, or other factors.

In the absence of high pain report, functional disability and psychological impairment, children with JIA show generalized alteration in pain threshold and sensory perception of all stimulus modalities compared to healthy controls. We followed the guidelines and QST protocol for children according to standardized procedures to reduce variation QST measures. Healthy control data were collected from two different countries and conducted by a range of experimenters and languages, and included the similar age range. There is the potential for subtle changes in QST data acquisition that may influence the variability of the data. We note that thermal testing in the JIA patients was conducted with a smaller thermode test area (1.6 × 1.6 cm) compared to the healthy controls (3 × 3 cm). The choice of probe was based on contact area required around relatively small joints. A pilot study performed in 8 healthy volunteers showed no trend in the direction of sensitivity (increased or decreased) when comparing the two probe sizes (data not shown). Indeed, one would predict that under normal conditions as stimulus area decreases, overall perceived cool or warmth intensity is likely to be lower (less sensitive) because the density of activated afferent fibers is smaller, which was contrary to our results. Additionally, a small number of children (n = 8; 2 with active arthritis, and 6 in clinical remission) were prescribed daily NSAIDs at the time of study that has the potential to dampen experimental pain perception [28]. In view of these factors, we still observe substantially marked pain hypersensitivity for pressure, heat and cold modalities, as well as sensory detection hyposensitivity for vibration, thermal cool and warm modalities for the entire sample of JIA patients compared to both healthy control groups.

### Persistent sensory alterations in pediatric chronic pain conditions

Our findings extend the current literature and suggest that children with JIA exhibit heightened pain sensitivity to multiple modalities compared to healthy controls. These results are consistent with other childhood chronic pain conditions investigating pain threshold, including complex regional pain syndrome [23], sickle cell disease [25], fibromyalgia [29], recurrent abdominal pain [30], tension-type headache and migraine [31, 32]. In addition to pain hypersensitivity, we also observed increased sensory detection thresholds (widespread hyposensitivity) to innocuous vibration and thermal detection modalities. These results are novel in this population and consistent with other studies examining recurrent pain in childhood. Work by McGrath’s group recently showed that children with sickle cell disease were less sensitive to thermal warm and cool temperatures, but hypersensitive to cold pain when compared with controls [30]. Children exposed to tissue (burn) injury during infancy are shown to have mechanical pain hypersensitivity and elevated mechanical detection thresholds (hyposensitivity) when tested at a non-burn site and compared to healthy controls [33]. Children who underwent cardiac surgery in infancy also exhibit mechanical hyposensitivity in a non-injured thenar eminence site compared to age-matched control [34]. The underlying mechanisms remain unclear but may be due to the fact that different afferent fibers types may be more susceptible to experience-dependent stress, pain exposure, joint damage, and analgesic and anti-inflammatory treatments [3].

The generalized enhanced sensitivity to mechanical and thermal stimuli modalities in joint areas, and those outside of the area of local inflammation, in children with JIA may reflect global changes in peripheral and central pain processing. Persistent inflammation at critical stages in early life, as seen in children with JIA, may modulate key sites involved in pain processing. At the periphery, enhanced release of pro-inflammatory cytokines, prostaglandins, peptides and growth factors from the inflamed tissue surrounding the joint that sensitizes nociceptor terminals [35]. Even in the absence of persistent inflammation, exposure to a mild physical insult such as visceral inflammation or repeated visceral distension which causes no lasting tissue damage, or a stressful environment in young rats leads to prolonged hypersensitivity of afferent nociceptive fibers [36, 37]. In addition, central sensitization of sensory circuitry, as characterized by increased neural excitability, strengthened excitatory input and decreased inhibitory activity is thought to be a key component in the transition from acute to chronic pain, and is hypothesized to play a role in pain in adult rheumatologic disease [38]. Finally, descending control from the brainstem, and functional connectivity changes at higher brain centers involved in pain processing at a critical time of synaptic plasticity may be implicated [3]. However, the role of central sensitization in these patients remains speculative, since overall pain reports were low.

### Limitations

QST was performed according to standardized instructions for children to ensure inter-experimental reliability. Nevertheless, QST measures can be influenced by a range of factors including experimental anxiety, attention, learning, coping style, and environmental conditions. Sensory thresholds in both normal subjects and in JIA patients vary according to sites tested. We evaluated joints in the JIA patients based on the locations of greatest past or present inflammation for each individual patient. Further QST profiling studies are needed to compare changes in sensory function at site-specific locations in larger samples for each involved joint, both in children with JIA patients and in healthy controls. Two historical datasets on subjects from two different countries, with larger sample sizes and a similar age range were used in this study for reference comparisons. While subtle changes in testing protocols and data capture methods may exist, it is remarkable how the changes in sensitivity seen in children with JIA are consistent when compared to both healthy control groups.

## Conclusions

We have demonstrated that while the majority of children with JIA in this study reported mild pain and mild impairment or disability, as a group, they showed measurable abnormalities in sensory processing compared to healthy controls. The changes in the sensory profile were detected even when little evident local or systemic inflammation was seen clinically. These results may be consistent with a pattern of central sensitization and highlight the necessity to improve the management of JIA with a multidisciplinary treatment approach. A small subset of JIA patients seen in our Rheumatology and Pain Clinics have greater degrees of pain and disability than most those in the current study. In some cases, pain and disability is present with comparatively mild indices of local or systemic inflammation. For these patients, it is plausible that treatment should emphasize not just suppression of inflammation, but also more broad-based approaches to management of pain. For example, it may be worth considering pharmacologic approaches to mitigate pain hypersensitivity (e.g. anticonvulsants, antidepressants and other medications used for neuropathic pain). In addition, cognitive-behavioral therapy, lifestyle interventions, physical therapy approaches, including active exercise, and other rehabilitative approaches that activate intrinsic pain inhibitory modulation. Future research may further analyze mechanisms underlying pain hypersensitivity in JIA, which will in turn guide pharmacologic and non-pharmacologic interventions to prevent or reverse these processes.

## Electronic supplementary material

Additional file 1: **QST Script.** (PDF 84 KB)

Additional file 2: **Table A: Absolute QST values in children with JIA and healthy controls at the thenar eminence.** (PDF 78 KB)

Additional file 3: **Table B: Linear regression analysis of predictors of QST data.** (PDF 84 KB)

## Abbreviations

CDT: Cold detection threshold
CPT: Cold pain threshold
FDI: Functional disability index
HPT: Heat pain threshold
JIA: Juvenile idiopathic arthritis
MDT: Mechanical detection threshold
MPT: Mechanical pain threshold
N: Newton
PedsQL: Pediatric quality of life inventory-multidimensional fatigue scale
PCS: Pain catastrophizing scale
PPT: Pressure pain threshold
QST: Quantitative sensory testing
T-STAI: Trait portion of the state-trait anxiety inventory
VDT: Vibration detection threshold
WDT: Warm detection threshold.
