# Supplementary material for: Pain hypersensitivity in juvenile idiopathic arthritis: a quantitative sensory testing study
Source: Pediatr Rheumatol Online J. 2014 Sep 6;12:39. doi: 10.1186/1546-0096-12-39 (PMC4171552; doi:10.1186/1546-0096-12-39)
Supplement: Supplementary file 1 — Additional file 1: QST Script. (PDF 84 KB) [file 12969_2014_2159_MOESM1_ESM.pdf]

Supplementary Material:

QST SCRIPT

Verbal instructions for performing quantitative sensory testing in children and adolescents (adapted from Blankenberg et al, 2010). The QST tests were performed in the order listed.

1. **Testing mechanical detection threshold (MDT).**

   "This is a test of your ability to detect light touch. I will press these hairs to your skin (specify area). Please tell me if you feel it or not."

2. **Testing mechanical pain threshold (MPT).**

   "This is a test of your ability to detect a 'sharp' sensation. I will continue to press these hairs gently on to your skin. If the hair feels like a 'soft' or 'hard' sensation, please say 'soft' or 'hard'. Please say 'sharp' immediately if it feels like a 'sharp' sensation."

3. **Testing vibration detection threshold (VDT).**

   "This is a test of your ability to detect vibration. I will put this vibration device on your skin (specify area). Please click the stop-button as soon as you start to feel vibration."

4. **Testing pressure pain threshold (PPT).**

   "This is a test of your sensitivity to pressure pain. I will press this pressure device against your skin (specify area). Please say ´Now´ as soon as the pressure starts to feel uncomfortable."

5. **Testing cold detection threshold (CDT).**

   "Please press the stop-button as soon as you feel the change in temperature." Once pressed, ask "What did you feel?" Repeat instructions again at random intervals once thermode returns to baseline temperature.

6. **Testing warm detection threshold (WDT).**

   "Now the temperature will go in the other direction. Please press the stop-button as soon as you feel the change in temperature ´. Once pressed, ask "What did you feel?" Repeat instructions again at random intervals once thermode returns to baseline skin temperature.

7. Testing cold pain threshold (CPT).

“The temperature of the skin will go down from ‘cool’ to ´cold´. Please press the stop button immediately as soon as it feels so cold you don’t want it on your skin anymore, like holding ice or a popsicle”

8. Testing of heat pain threshold (HPT).

“The temperature of the skin will go up from ´warm´ to ´hot´. Please press the stop button as soon as the temperature feels so hot you don’t want it on your skin anymore, like holding a cup of hot coffee or hot chocolate”.
