# Supplementary material for: Pain hypersensitivity in juvenile idiopathic arthritis: a quantitative sensory testing study
Source: Pediatr Rheumatol Online J. 2014 Sep 6;12:39. doi: 10.1186/1546-0096-12-39 (PMC4171552; doi:10.1186/1546-0096-12-39)
Supplement: Supplementary file 2 — Additional file 2: Table A: Absolute QST values in children with JIA and healthy controls at the thenar eminence. (PDF 78 KB) [file 12969_2014_2159_MOESM2_ESM.pdf]

Supplementary Table A: Absolute QST values in children with JIA and healthy controls at the thenar eminence[a].

QST parameter	Control-US (n=92)	Control-EU (n=151)	JIA (n=60)[b]	*p-value*[c]
**Innocuous Stimulus**				
MDT (g)	---	0.23 (0.18-0.33)	0.04 (0.28-0.07)	**<0.0001**
VDT (um/sec)	0.2 (0.2-0.25)	---	0.68 (0.53-0.90)	**<0.001**
CDT (°C)	30.6 (30.0-30.9)	31.1 (30.6-31.3)	30.2 (29.5-30.6)	**US: <0.01**<br>**EU: <0.001**
WDT (°C)	33.7 (33.4-34.1)	33.4 (33.1-34.0)	34.8 (34.1-35.5)	**US: <0.0001**<br>**EU: <0.0001**
**Noxious Stimulus**				
PPT (N)	---	45.8 (35.6-55.6)	10.3 (6.3-15.5)	**<0.001**
CPT (°C)	13.8 (5.1-21.4)	21.6 (13.4-25.2)	25.6 (17.3-28.9)	**US: <0.001**<br>**EU: <0.01**
HPT (°C)	42.3 (39.6-47.2)	40.6 (38.1-43.9)	38.2 (36.0-42.3)	**US: <0.001**<br>**EU: <0.05**

Children with JIA have increased sensitivity to touch, pressure, cold and heat pain compared to healthy controls.

CDT, Cold Detection Threshold, CPT, Cold Pain Threshold; EU, European control; HPT, Heat Pain Threshold; IQR, Inter-Quartile Range; MDT- Mechanical Detection Threshold; MPT, Mechanical Pain Threshold; PPT, Pressure Detection Threshold; US, US control; VDT, Vibration Detection Threshold; WDT, Warm Detection Threshold.

[a.] All values are given as median (IQR). [b.] 60 JIA patients included in the analysis except vibration (n=53), thermal (n=56) and pressure (n=58) stimulus modalities where data was not available due to technical issues. [c.] *p*-values indicate differences in sensory threshold between Control and JIA groups. Mann-Whitney *U*-test and Kruskal-Wallis with Dunn's post-hoc test used where appropriate.
