# Supplementary material for: Pain hypersensitivity in juvenile idiopathic arthritis: a quantitative sensory testing study
Source: Pediatr Rheumatol Online J. 2014 Sep 6;12:39. doi: 10.1186/1546-0096-12-39 (PMC4171552; doi:10.1186/1546-0096-12-39)
Supplement: Supplementary file 3 — Additional file 3: Table B: Linear regression analysis of predictors of QST data. (PDF 84 KB) [file 12969_2014_2159_MOESM3_ESM.pdf]

Supplementary Table A: Linear regression analysis of predictors of QST data.

	Noxious stimulus								Innocuous stimulus							
QST predictor	MPT		PPT		CPT		HPT		MDT		VDT		CDT		WDT	
	r^2	p	r^2	p	r^2	p	r^2	p	r^2	p	r^2	p	r^2	p	r^2	p
**QST site**																
Upper vs. lower extremities	0.014	0.363	0.044	0.114	0.007	0.530	0.087	**0.028**	0.515	0.081	0.351	**0.000**	0.225	**0.000**	0.168	**0.002**
**Demographics**																
Gender	0.016	0.767	0.058	0.363	0.215	**0.000**	0.115	0.194	0.085	0.156	0.352	0.794	0.229	0.749	0.170	0.733
Age	0.065	0.100	0.121	0.032	0.050	0.129	0.088	0.809	0.078	0.202	0.384	0.110	0.228	0.848	0.177	0.438
**Markers of disease activity**																
Duration of rheumatology clinic attendance	0.030	0.342	0.108	0.052	0.009	0.300	0.102	0.352	0.517	0.915	0.368	0.252	0.230	0.681	0.188	0.252
No. of active joints	0.024	0.465	0.044	0.976	0.097	0.026	0.111	0.237	0.056	0.060	0.352	0.833	0.242	0.328	0.206	0.118
Erthroctye Sedimentation Rate	0.006	0.928	0.024	0.691	0.005	0.655	0.051	0.827	0.051	0.926	0.332	0.533	0.207	0.703	0.147	0.817
C-HAQ score	0.029	0.622	0.062	0.617	0.003	0.951	0.046	0.948	0.194	0.186	0.363	0.396	0.263	0.274	0.204	0.401
**Pain**																
Patient pain assessment	0.047	0.199	0.050	0.305	0.009	0.573	0.073	0.761	0.132	0.706	0.347	0.657	0.236	0.790	0.162	0.833
Pain Frequency	0.026	0.476	0.080	0.102	0.007	0.634	0.095	0.267	0.133	0.636	0.345	0.789	0.246	0.420	0.169	0.510
**Psychological Function**																
FDI	0.026	0.418	0.045	0.816	0.034	0.234	0.120	0.160	0.053	0.784	0.351	0.863	0.294	0.030	0.187	0.270
PCS	0.033	0.302	0.085	0.121	0.010	0.704	0.131	0.106	0.062	0.138	0.361	0.378	0.231	0.654	0.170	0.716
Pediatric Symptom Checklist	0.018	0.645	0.048	0.648	0.060	0.092	0.147	0.057	0.055	0.672	0.362	0.348	0.276	0.066	0.175	0.488
T-STAI	0.117	**0.013**	0.046	0.720	0.030	0.277	0.148	0.055	0.115	0.048	0.351	0.943	0.244	0.298	0.177	0.438
PedsQL	0.023	0.562	0.104	0.062	0.008	0.553	0.096	0.549	0.151	0.244	0.358	0.630	0.320	**0.012**	0.179	0.766

Individual linear regression analyses in the arthritic joint were applied for each QST measure to evaluate possible predictors. Results are expressed as estimated region coefficients (r^2) in each predictor value, and significance level (p-value). Bonferroni correction was used and p-values equal or <0.01 were considered significant. Bold typeface indicates significant values.

CDT – Cold Detection Threshold; CPT – Cold Pain Threshold; FDI – Functional Disability Index; HPT – Heat Pain Threshold; JIA – Juvenile Idiopathic Arthritis; MDT – Mechanical Detection Threshold; MPT – Mechanical Pain Threshold; PedsQL – Pediatric Quality of Life inventory-Multidimensional Fatigue Scale; PCS – Pain Catastrophizing Scale; PPT – Pressure Pain Threshold; QST – Quantitative Sensory Testing; T-STAI – Trait portion of the State-Trait Anxiety Inventory; VDT – Vibration Detection Threshold; WDT- Warm Detection Threshold.
